# Arazá: *Eugenia stipitata* Mc Vaught as a Potential Functional Food

**DOI:** 10.3390/foods13152310

**Published:** 2024-07-23

**Authors:** Luis Acosta-Vega, Diego A. Moreno, Liceth N. Cuéllar Álvarez

**Affiliations:** 1 Grupo de Investigación en Productos Naturales Amazónicos-GIPRONAZ, Universidad de la Amazonia, Florencia 180001, Colombia; lh.acosta@udla.edu.co; 2 Laboratorio de Fitoquímica y Alimentos Saludables (LabFAS), CEBAS, CSIC, Campus Universitario de Espinardo, 25, E-30100 Murcia, Spain; dmoreno@cebas.csic.es

**Keywords:** functional food, bioactive compound, amazonian fruit, biological activity

## Abstract

Arazá is a fruit native to the Amazonian region with characteristic properties such as aroma, texture, color, and marked acidity. Additionally, the fruit is rich in bioactive compounds in its three fractions (seed, pulp, and peel), such as ascorbic acid, phenolic compounds (and their derivatives), and carotenoids, which have been extensively investigated in the literature for their beneficial properties for human health. However, it is a little-known fruit, and the role it can play in health-promoting activities related to the treatment and prevention of non-communicable diseases (NCDs) when incorporated into the diet is also unknown. Therefore, it is necessary to know the profile of bioactive compounds and the biological properties Arazá possesses, which is the aim of this review.

## 1. Introduction

Recently, there has been a significant shift towards adopting healthy habits and lifestyles, where an active routine and consciously consuming minimally processed or unprocessed foods of a natural origin have increased over recent years. This shift is primarily driven by the growing concern over non-communicable diseases (NCDs), which are now a major global health issue associated with degenerative conditions like cancer, diabetes, obesity, and cardiovascular, neurological, and respiratory disorders [1,2]. These diseases are responsible for a staggering number of deaths worldwide each year. Among the various risk factors for NCDs, behavioral modifications, such as regular physical activity and a diet rich in nutrient-dense foods, including functional foods, are crucial in reducing the risk of developing these diseases [3,4].

What does functional food mean? Functional foods are generally defined as those than provide more than simple nutrition. They supply substances that offer benefits to the consumer. Functional foods are similar in appearance or even are conventional foods that can be included in a practical way in the diet and even have shown to provide compounds with beneficial biological activities, such as disease prevention or recovery, in addition to fulfilling nutritional requirements [5,6]. Also, it is essential to mention that a food can also be considered functional when modified and transformed by different processes to increase its beneficial effect on human health. However, the functional foods are not those designed to treat a disease, to make up for nutritional deficiencies, or to be an isolated/purified fraction of food that has shown beneficial effects in treating chronic diseases [7]. Functional foods are characterized by being rich in bioactive compounds, especially those of an antioxidant nature, including phenolic compounds (PC), ascorbic acid (AA), and carotenoids (C). Those compounds regulate oxidative/high-energy species produced in cellular environments during oxidative stress and prevent DNA and protein damage that can cause dysfunction at the cellular level and lead to mutations, genetic instability, and epigenetic modifications [8], which are strongly related to the apparition of NCDs [8].

Therefore, one of the main challenges of the food industry is the development of functional foods with a high content of bioactive compounds that demonstrate biological activities (such as an antioxidant capacity). Additionally, searching for new flavors, smells, textures, and unconventional characteristics is a driving aspect of novel food product developments where biodiversity in Amazonian fruit species may serve as a basis for obtaining innovative new functional foods with exotic appearances enriched in bioactive compounds of interest [9,10]. Among them, Arazá (*E. stipitata*), also called araça-boi, is a fruit species native to the Amazon, cultivated in Ecuador, Colombia, Brazil, Peru, and Bolivia. *E. stipitata* have a flattened-to-round morphology, an average weight of 30–80 g, few seeds, a thin peel, and an intense yellow color when ripe. The pulp is fleshy and highly acidic and, consequently, is not consumed in nature, but is used to prepare beverages, candy or sweets, ice creams, and cocktails, among others [11]. The fruit’s characteristic aroma and unique flavor make it a potential raw material for food processing [12].

Arazá belongs to the Myrtaceae family, which is composed of more than a hundred genera and a large number of species, including guava (*Psidium guajava* L.), jaboticaba (*Myrciaria cauliflora*), and pitanga (*Eugenia uniflora* L.). Those fruits are commercially exploited and are economically important crops, while *E. stipitata* is not [13,14]. Among the related studies, the presence of bioactive compounds and the antioxidant activity of the fruit can enhance its use as a possible functional food, promoting strategies for extending the recognition of the fruit and its commercial exploitation [15]. Therefore, to consider Arazá as a functional food, it is necessary to evaluate and assess the content of the bioactive compounds present in the fruit and the different biological functions they may have. This review aims to provide information on the content of the bioactive compounds and the biological activities reported for Arazá and its parts (pulp, seed, and peel) that may allow the fruit to be considered a functional food in preventing or developing NCDs.

## 2. Materials and Methods

The literature reviewed consist of articles written in English and Spanish published between January 2000 and May 2023 in peer-reviewed journals. The databases for retrieving articles were Science Direct, Google Scholar, Wiley, Scopus, SciFinder, SpringerLink, and Web of Science. The keywords used for searching were “Eugenia stipitata” AND bioactive”, “Eugenia stipitata” AND “food” OR “bioactive” OR “functional”. Given the limited recognition of the fruit, and to retrieve as much information as possible, the search equation was modified, replacing “Eugenia stipitata” with “araçá-boi” and “Arazá”. The systematic review’s reporting followed the PRISMA statement’s standards, and the selection criteria and workflow can be found in the Supplementary Material.

## 3. Results

A total of 36 articles were included in the analysis of the present review. This work is composed of two main sections: the first one centers its attention on the content of bioactive compounds and the organoleptic aspects of the fruit, and the second part is the potential biological activity or functional properties according to their interaction between the presence of bioactive compounds in seeds, peel, and pulp, depending on the physiological state (ripening), geographical location, and processing practices, in connection with the prevention of NCDs.

### 3.1. Botanical Aspects and Economic Importance

Arazá (also known as araçá-boi in Brazil and membrillo in Ecuador) is a fruit belonging to the Myrtaceae family and the genus Eugenia, within which approximately 400 species have been described, including *Eugenia uniflora* L., *Eugenia involucrata*, *Eugenia pyriformis* Cambess, and *Eugenia desinterica* DC, which are fruit-producing plants whose potential use as sources of secondary metabolites with biological activities and raw materials for the development of food products has been explored [11,13,16,17]. This is not the case with our study species (*Eugenia stipitata* Mc Vaught), which has been explored and studied to a lesser extent. It is a native fruit of the Amazon region and is distributed in Brazil, Colombia, Peru, Bolivia, and Ecuador. It grows on medium-sized trees (2.5–15.0 m) with highly branched crowns. The fruit is climacteric and is harvested three times a year; its morphology varies from globose to a spherical berry with an average diameter of 12 cm and can weigh up to 80 g. It has a thin layer of peel with a smooth surface that varies from a pale green when it is immature to a bright yellow when fully ripe. On the inside, it has seeds ranging from 4–10 per fruit, surrounded by a juicy, white–yellow, fleshy, and very aromatic pulp, the latter being the edible part of the fruit. However, the pulp is highly acidic, so it is not generally consumed raw; it is used in various food preparations, such as juices, ice cream, and jellies [14,18,19,20,21].

The high acidity of the pulp is mainly attributed to the pH (pH = 2.4), also, the pulp has a high moisture content (>90%) and a low lipid content (<0.4%); in dry weight, the pulp is mainly composed of sugars (49.2%), with fructose being the significant proportion, dietary fiber, amino acids, vitamins, and minerals [22,23]. The peel is rich in phytochemicals (especially phenolic acids derivatives) [10,18], and the seeds, as well as the pulp, are rich in sugars and bioactive compounds such as phenolic compounds and their derivatives, carotenoids and vitamins [14,24]. Therefore, the fruit of *Eugenia stipitata* can be considered a source of bioactive compounds and other metabolites with nutritional value that can be included in the diet to take advantage of the biological activities they offer. However, the limited research on this fruit coincides with several authors’ statements that the fruit needs to be adequately exploited [14,18,21,25,26]. In addition, the marketability of the fruit is low compared to conventional fruits. It is mainly unknown worldwide and even in regions of countries where it is cultivated as it is especially conditioned to local shops (of which it is one of the primary sources of income for rural families) and small enterprises oriented to the production of food products [22]. Therefore, given the potential Arazá represents as a native fruit of the Amazon, there is a need to direct research in the direction of extensively exploring the functional properties that its consumption can provide, which can be exploited by incorporating it through its processing at an industrial level. However, first, it is necessary to know the trends in knowledge and the existing perspectives on Arazá to disseminate the future applications that this fruit can have.

### 3.2. Bioactive Compounds in Arazá

The content of bioactive compounds in plant-based foods has gained increasing interest in recent decades from society in general due to the beneficial effects that can be generated in people’s life quality. Among the main positive effects, decreasing the risk of chronic diseases such as NCDs and even contributing to improving patient conditions and recovery are the main reasons related to the inclusion of bioactive compounds in people’s diets [27,28]. Additionally, their natural origin makes them valuable substances since they can be extracted from different plant sources, i.e., leaves, stems, barks, seeds, and fruits [29]. The bioactive compounds are generally secondary metabolites, and their function within the target organism is not oriented towards the nutritional role, but towards the biological activity on which they have an effect; therefore, keeping in account the existent biodiversity, it can be stated that there is a plethora of compounds with biological activity [30]. However, it is worth mentioning that the existence of hundreds of different chemical compounds in natural samples that have demonstrated biological activity previously requires screening, isolation, and further analysis to obtain insights about the compounds responsible for the biological activity observed [31,32]. 

Due to the great interest in this type of metabolite, countless tests and experiments have been carried out to elucidate and establish the different functionalities that those metabolites can exert. This allows us to know in a specific way the metabolites and group of metabolites that met the previous definition [28,33,34,35,36]. In the case of the present study, ascorbic acid, phenolic compounds, and carotenoids are the main bioactive compounds present in the peel (epicarp), the pulp (mesocarp), and the seeds of Arazá (Table 1). Factors such as the ripening stage, edaphoclimatic conditions, and origin influence the content and variations of these metabolites [10,37]. 

**Figure 1:**
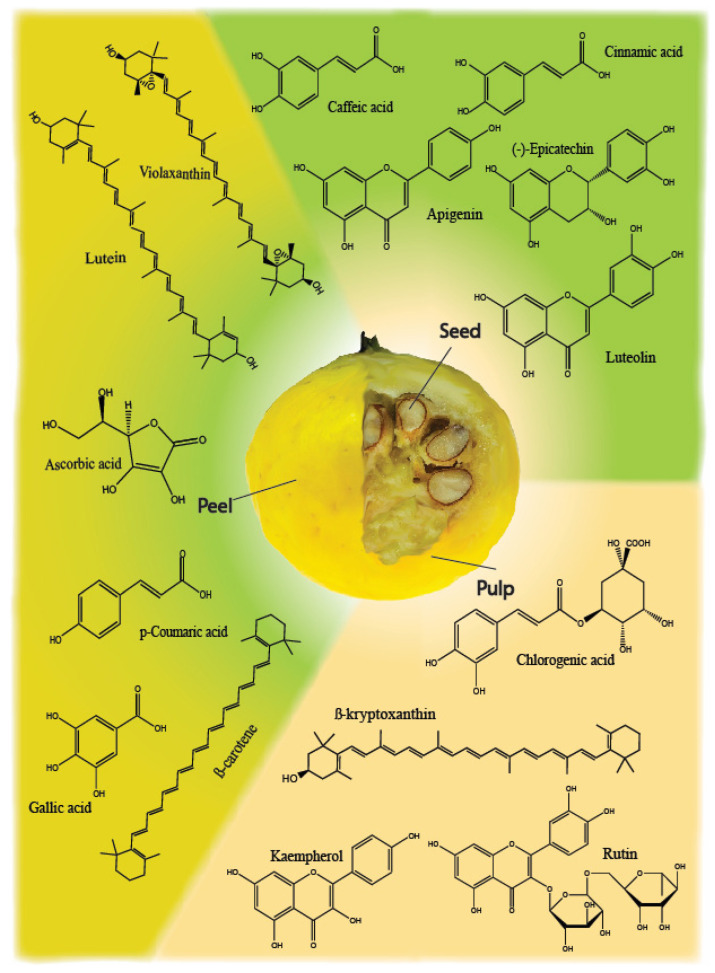
The main bioactive compounds present in Arazá’s different parts.

Furthermore, the reported transformations to which the fruit (and its parts) has been subjected to preserve its beneficial properties and produce an alternative to its consumption also affect the composition and quantity of these metabolites. Furthermore, it is essential to highlight that the extraction method is a substantial aspect that significantly affects the stability of these compounds and the nature of the extract obtained. In the present case, evidence is given regarding the use of organic solvents for the extraction of the different compounds, and these are consequently analyzed using spectrophotometric techniques and liquid chromatography (LC-MS) for both quantitative and qualitative purposes. Each of these steps involves the advantages and disadvantages that condition one’s view of the fruit, and therefore, it is necessary to carry out an adequate experimentation performance that allows a better understanding of these metabolites in the fruit and the dynamics related to their preservation, transformation, and characterization for possible future applications. The primary bioactive compounds found in *E. stipitata* are depicted in Figure 1 and are thoroughly described in the subsequent sections.

#### 3.2.1. Vitamin C

The interest in vitamin C (ascorbic acid) has been ongoing for several decades, as it is an indispensable nutrient obtained exclusively from the diet [38]. It is involved in various metabolic processes and plays an active role in the function of different parts of the body, such as the normal functioning of the immune system and collagen production, it is a cofactor in the absorption of other nutrients, and it shares its antioxidant capacity with its oxidation product (dehydroascorbic acid) [39]. In addition, it has recently been shown that vitamin C can have a positive effect as a supplement in the recovery and control of NCDs such as obesity, diabetes, and cardiovascular and pulmonary diseases [40]. According to the reports retrieved in the present work, there are few reports on the ascorbic acid content of the Arazá fruit, which have mainly focused on the pulp (127.8 mg/100 g) and peel (184.8 mg/100 g), while for the seeds, there are no reports of this antioxidant (Table 1). Given the labile nature of this substance, research has focused on monitoring the content of this substance through the different transformations to which it can be subjected, or even just its post-harvest behavior, as in the case of the study carried out with Arazá fruits harvested in the department of Caquetá (Colombia), where a decrease of approximately 22% of ascorbic acid was observed in the pulp after twelve days of ripening (without being harvested) [37]. Similarly, the same post-harvest behavior was observed in fruits from the northern Amazonian region of Brazil, as the content of this metabolite decreased by about 80% for the peel and 90% for the pulp after 12 days of storage [10]. Variations in the content of this metabolite in fruits are mainly attributed to environmental factors (light, drought, heat, and salts present in soil) since this metabolite plays an essential role in the electrochemical balance of the fruit, as well as its resistance to stress, fruit development, and post-harvest behavior [39,41,42].

The application of different treatments in fruit parts influences the ascorbic acid content. For example, the application of high-intensity ultrasound to Arazá’s pulp affected the pH, total soluble solids, and the content of this antioxidant; the results showed that the application of an ultrasound of high energy (U7000 treatment with an energy density of 7000 J/g at a power of 400 W) reduced the pH and increased the content of total soluble solids and vitamin C (+16.0% and +46.51%, respectively) significantly (*p* < 0.05) concerning untreated pulp. This is attributed to cell lysis due to the cavitation effect generated by the sonication, which promotes the release of matrix compounds [43]. The impact of applying pasteurization to Arazá pulp has also been reported [44]; here, it was observed that processing at 65, 73, and 80 °C decreased the ascorbic acid content by 2, 4, and 12%, respectively. Furthermore, in the same study, it was observed that, in general, pasteurization contributed to a decrease in the loss of vitamin C during frozen storage, where the highest temperature used (80 °C) preserved 82.9% of the initial content after two months, indicating that the treatment applied increased the half-life of the frozen pulp, which is related to the inactivation of enzymes involved in the degradation of ascorbic acid [44]. Similarly, the effect of pasteurization treatment combined with sucrose before frozen storage on the ascorbic acid content was also evaluated, where a positive impact of both heat treatment (H) and heat treatment combined with sucrose (SH) was observed, extending the half-life of the frozen pulp from 93 days (control) to 166 days for H and 278 days for SH, as well as preserving its sensory characteristics (color, aroma, flavor, and texture) [45].

Given the heat-sensitive nature of ascorbic acid, it is necessary to use techniques that avoid high temperatures to prevent the degradation of this antioxidant, such as freeze-drying [46]. The stability of ascorbic acid in freeze-dried Arazá pulp powders were combined with two wall materials, gum arabic (AAG) and maltodextrin with ten dextrose equivalents (AMD), and was evaluated [47]. The encapsulating agent greatly influenced the preservation of ascorbic acid in the powders, with loss values ranging from 6–9%, with AMD having the lowest values. In addition, AAG had a longer half-life of more than 200 days, while AMD had a half-life of about 178 days [47]. On the other hand, the enrichment of apple nectars with freeze-dried Arazá pulp was evaluated. A modest increase in the vitamin C content in apple nectars was observed without significant modifications to the sensory characteristics [26]. 

**Figure 2:**
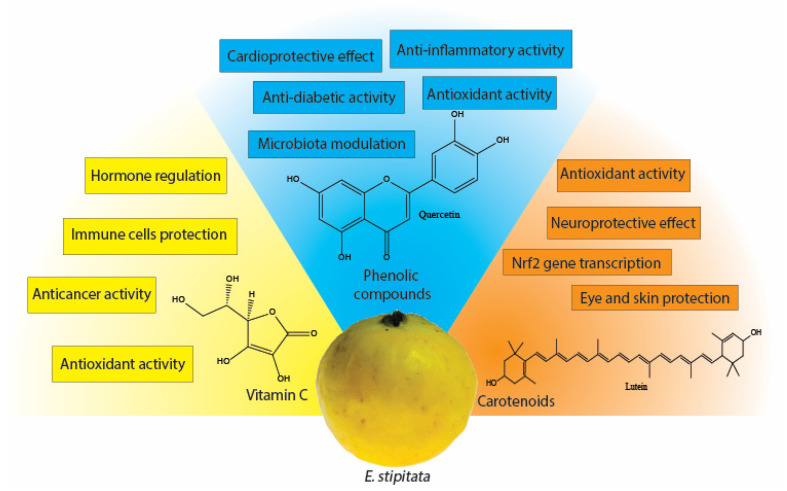
Functional biological activities of bioactive compounds present in Arazá.

Due to the above, the different processing techniques contribute to preserving substances with health benefits, such as ascorbic acid, and allow their addition to other food preparations without altering the final product’s qualities or affecting consumers’ acceptance perception. This is greatly important due to ascorbic acid’s benefits in treating NCDs. The evidence suggests that the risk reduction of developing neurodegenerative diseases, cancer, viral infections, and age-related illnesses is related to its role as an antioxidant, which limits and controls the radical and oxidant species produced in the cellular environment [4,48,49,50,51]. Additionally, it is involved in different physiological mechanisms, where it plays essential roles in hormonal regulation, central nervous system protection, and immunological cell stimulation. The concentration of ascorbic acid has been related as an indicator in the preliminary stages before the development of metabolic disorders and pre-diabetes [49,52,53,54,55] (Figure 2). 

#### 3.2.2. Phenolic Compounds

Phenolic compounds are secondary metabolites widely distributed in nature; their base structure consists of an aromatic ring to which at least one hydroxyl group is attached, and they can vary in structure and substituent complexity. They are usually classified according to the structures formed by the carbon atoms and are found naturally as organic acids or bonded to sugars. [56]. Additionally, there is also an internal classification within this group of substances, being of two types: flavonoids (with an intern classification) and the non-flavonoid type (phenolic acids, coumarins, xanthones, and stilbenes, among others). Flavonoids occupy about 2/3 of phenolic compounds, while the remainder corresponds mainly to phenolic acids and derivatives [57]. The growing interest in these compounds lies in the different biological activities demonstrated through vast scientific research, including in vivo and in vitro assays, where some of the leading natural components are extensively described to a greater extent [58]. Phenolic compounds possess a wide range of biological activities. Among them, their antioxidant, anti-diabetic, anti-cancer, and anti-inflammatory activity stands out, as well as their role in the prevention of cardiovascular diseases, anti-obesity, and neurodegenerative diseases, especially those of the central nervous system, which are closely related to the effects of aging and unhealthy lifestyles [59,60,61].

The total content of phenolic compounds in Arazá’s parts has been estimated in several studies, as shown in Table 1. The range of phenolic compounds in the seeds corresponds to 130.6 mg GAE/g d.w [12], 99.16 ± 0.79 mg GAE/g in the pulp [47], and 287.7 mg GAE/g in the peel [10]. On the other hand, the flavonoid content follows a similar trend in which the content is higher in the seeds than in the pulp, with a content of 24.1 mg CE/g d.w in the seeds [12], 6.07 ± 0.22 mg CE/g d.w in the pulp [62], and no reports were found exclusively for the peel, the closest being the one reported for the mixture between the pulp and seeds (9.7 mg CE/g d.w) [12]. Other values of the phenolic compounds and flavonoids are shown in Table 1. 

In the evaluation of the phenolic profile in freeze-drying Arazá powders by liquid chromatography, Reyes-Álvarez et al. [47] identified and quantified different phenolic compounds, including caffeic, chlorogenic, cinnamic, coumaric, gallic, and trans-ferulic acid (187.87, 166.98, 288.95, 354.23, 166.02, and 524.68 mg/100 g d.w, respectively), as well as flavonoids like eriodictyol (581.21 mg GAE/100 g d.w) quercetin (699.75 mg ruthinoside trihydrate equivalent/100 g d.w), and rutin (171.81 ruthinoside trihydrate equivalent/100 g d.w) [47]. An important aspect related to the inclusion of this fruit into the diet is its bioavailability in the intestinal tract for absorption since the digestive process can degrade the content of these and even alter their composition. For this reason, De Araujo et al. evaluated the bioavailability of phenolic compounds from Arazá [12]. Initially, nine flavonoids (apigenin, catechin, gallocatechin, kaempferol, luteolin, and quercetin) and eight phenolic acids (caffeoyl, coumaroyl, fertaric, gallic, and vanillic acids) were identified in the edible part. In comparison, ten flavonoids (apigenin, catechin, luteolin, myricetin, quercetin, and kaempferol) and seven phenolic acids (cinnamic, fertaric, gallic, and vanillic acids) were reported in the seeds. The results showed an increased flavonoid content in both fractions during the digestion and gastrointestinal phases. At the same time, the phenolic acids decreased in intensity, indicating the possible degradation of these compounds [12]. Similarly, Soares et al. evaluated the phenolic profile of the Arazá pulp [63], identifying compounds such as casuarictin I (and it is derivatives I, II, III, IV, and V), coumaric acid (hexoside), epicatechin, ellagic acid (hexoside), gallocatechin gallate, vanillic acid (hexoside), dihydroquercetin (glycoside), tellimagrandin II, epicatechin gallate, eriodictyol (glycoside), and pinoresinol (glycoside) [63].

The fruit’s ripening stage also influences the phenolic compounds’ content in the different parts of the Arazá fruit. This was evidenced in the study carried out by Cuellar et al. [64], where they evaluated four different ripening stages (green, semi-ripe, ripe, and overripe), finding that the content of the phenolic compounds measured by the Folin–Ciocalteu method is vastly higher in the epicarp than in the mesocarp, following the trend green > ripe > semi-ripe > overripe. In contrast, the trend observed in the mesocarp was green > semi-ripe > ripe > overripe. In the same study, the chlorogenic, caffeic, and gallic acid content was quantified in both fractions, reaching the maximum values of chlorogenic acid in the semi-ripe and overripe states. Conversely, caffeic acid reached the highest value in the mature and semi-ripe states for the epicarp and the mesocarp, respectively. Additionally, the highest value of gallic acid was observed in the overripe state for both parts. Similarly, in the research carried out by Galeano and collaborators [65], where they evaluated the content of phenolic compounds in five different stages of ripening: ripe green, semi-ripe, advanced semi-ripe (¾), mature, and overripe, the results of the content of phenolic compounds were as follows 346.1 ± 28, 630.1 ± 41, 1063.9 ± 23, 916.2 ± 18, and 383.5 ± 50 mg GAE/100 g dry extract, respectively.

Although the pulp is almost the only part of the fruit used, the seed and the peel (both considered agro-industrial by-products) can be sources of bioactive compounds. In the study carried out by Chagas-Barrios et al. [18], both fractions (mixed) were studied. A phenolic compound content of 42.81 ± 2.23 and 41.59 ± 6.26 mg/100 g and flavonoid content of 2.52 ± 0.25 and 2.52 ± 0.30 mg/100 g for the aqueous and methanolic extract were obtained, respectively. Additionally, other phenolic compounds were quantified, such as caffeic acid (0.46 ± 0.06 µg/g), cinnamic acid (44.61 ± 0.76 µg/g), *p*-coumaric acid (1.57 ± 0. 15 µg/g), (-)-epicatechin (18.05 ± 5.35 µg/g), ethyl gallate (0.37 ± 0.02 µg/g), ferulic acid (0.44 ± 0.02 µg/g), gallic acid (30.18 ± 5.47 µg/g), quercetin-3-glucoside (2. 47 ± 0.54 µg/g), and vanillin (0.09 ± 0.00 µg/g) in the aqueous extract. Furthermore, the content of these bioactive compounds is influenced by the nature of the solvent used, with aqueous extraction providing the highest amount of these compounds, demonstrating the potential use of these by-products as raw materials as a source of phenolic compounds. 

On the other hand, the fruit is not generally consumed raw; it is processed due to its strong acidity. Baldini et al. [26] prepared apple nectars supplemented with freeze-dried (freeze-drying) Arazá pulp, which increased the content of phenolic compounds and flavonoids in the commercial nectars compared to the control without altering the sensory perception. In the same way, the use of other techniques such as spray-drying, high-intensity ultrasound, pasteurization, and heat treatments have been shown to contribute effectively to the preservation of these metabolites, transforming them and guaranteeing a longer half-life compared to the fruit after harvesting [43,44,45,66]. 

The application of different processing techniques in the Arazá is needed due to the wide range of benefits of phenolic compounds in treating and preventing NCDs. Phenolic acids (such as caffeic, gallic, ellagic, and chlorogenic acid), for example, have been shown to have anti-diabetic effects through different mechanisms, such as the inhibition of the activity of α-glucosidase and α-amylase, as well as the inhibition of glucose production in hepatic cells, which is reflected in a lower glucose level in plasma, which indirectly affects insulin sensitivity [67,68,69,70,71]. Similarly, flavonoids such as quercetin, rutin, and kaempferol have shown anti-diabetic effects as well as microbiota modulation, which are reflected in changes in glycan degradation and fatty acids metabolism, which highly depend on the phenolic compounds’ concentration and bacteria strain present [72,73,74]. On the other side, the anticancer, anti-inflammatory, and protective effects of phenolic compounds are generally attributed to the antioxidant activity of these compounds and their signaling functions in the interaction with different cellular mechanisms, such as the neutralization of reactive oxygen species (ROS), immune cells’ activation, the regulation of enzyme antioxidant systems, cell migration, and the inhibition of fatty acids’ metabolism [75,76,77,78,79,80,81] (Figure 2).

#### 3.2.3. Carotenoids

Carotenoids are chemical polyene-type compounds involved in the photo-protection of plants and precursors for the biosynthesis of phytohormones and other signaling molecules [82]. They are classified into two groups: carotenes and xanthophylls. The first group comprises only substances composed of carbon and hydrogen atoms, while the second group consists of oxygenated structures of carotenes [83]. Since they are exogenous nutrients, the primary role of these compounds in human nutrition is related to their antioxidant power, especially against reactive oxygen species, which, when produced in excess and not controlled, can lead to oxidative stress, which is considered one of the main contributors of pathogenic processes of different degenerative diseases, such as cardiovascular, neurodegenerative or vision-related disorders [84]. Few investigations have studied the carotenoid profile of Arazá, and even for the present work, no reports were found on the carotenoid content in the seed of Arazá, as the reports are exclusively oriented to the pulp and peel of the fruit. The highest carotenoid content in the pulp is 62.85 ± 1.92 µg β-carotene/g and in the peel, 24.84 µg/100 g f.w (Table 1). 

Within the carotenoid profile of the pulp, a wide variety of carotenoids (alpha and beta) are found: *β*-kryptoxanthin, 9-cis-*β*-carotene, 13-cis-*β*-carotene, 15-cis-*β*-carotene, Violaxanthin, Lutein, Zeaxanthin, and Zeinoxanthin [21,85]. Additionally, in vitro digestion tests show that the carotenoids in both fractions have more than 15% bioavailability, with zeaxanthin, 15-cis-*β*-carotene, and All-trans-*α*-carotene retaining the highest proportion. The most abundant carotenoid in both the pulp and the peel is lutein (including all its esterified forms). The presence of zeaxanthin in both parts of the fruit is particularly interesting because these two carotenoids are related to the reduction of irreversible blindness in older adults and other problems related to vision in the older population [86]. Finally, the ripening stage influences the composition of carotenoids in the pulp of Arazá, with the green stage rich in lutein and *β*-kryptoxanthin, the medium ripening stage (half green) consisting mainly of *β*-carotene and lutein esters, and the mature stage consisting mainly of lutein (in free form and esters) as well as zeinoxanthin and *β*-kryptoxanthin esters [21,85].

One of the most remarkable aspects of carotenoids is their antioxidant activity, especially as a single oxygen quencher in the cellular redox signaling pathway [83]. Additionally, their chemical structure allows them to interact with other antioxidants on lipophilic substracts, such as tocopherols, which also enhance their antioxidant efficiency in the propagation of other radical species, such as nitrogen-derived species and fatty acid peroxides [87,88]. Conversely, carotenoids regulate the expression of genes encoding antioxidant enzymes through Nrf2 gene transcription, acting as indirect antioxidants in oxidative stress control [89,90]. These mechanisms are reflected in the protective effects and treatment of peel and eye photo-degradation and chronic diseases like cardiovascular disease and cancer [91,92,93]. However, the carotenoid intake is still a subject of discussion due to its accumulation in mammals, which can induce pro-oxidant activity during lipid oxidation [94] (Figure 2).

**Table 1:** Metabolite and bioactive compound content reported for Arazá.

Metabolite	Fruit Part	Composition	Units	Reference
Phenolic compounds (total)	Freeze-dried pulp	99.16 ± 0.79	mg GAE/g d.w	[47]
		184.8 ± 8.25	mg GAE/g	[95]
		19.3 ± 5.1	mg GAE/g f.w	[21]
		1569.60 ± 3.31	mg GAE/100 g d.w	[62]
		1569.60 ± 3.31	mg GAE/100 g d.w	[62]
		286.7 ± 14.67	mg GAE/100 g d.w	[64]
		3507.79 ± 13.97	mg GAE/100 g	[96]
	Pulp + peel	31.2	mg GAE/g d.w	[12]
		9.06 ± 0.42	mg GAE/100 g d.w	[14]
		35.7	mg GAE/g f.w	[15]
	Seeds	130.6	mg GAE/g d.w	[12]
		142.43 ± 0.82	mg GAE/100 g d.w	[14]
		16.6 ± 0.8	mg GAE/gTS	[9]
	Pulp	1.57 ± 0.0	mg GAE/g	[97]
		275.4	mg GAE/g d.w	[10]
		30.16 ± 0.7	mg GAE/gTS	[9]
		122.78 ± 2.52	mg GAE/100 g	[98]
		87 ± 2	mg/100 g f.w	[99]
		51.91 ± 0.12	mg GAE/100 g	[100]
		1063.9 ± 23	mg GAE/100 g d.w	[65]
		287.7	mg GAE/g d.w	[10]
	Spray-dried pulp	2930 ± 50.0	mg GAE/g d.w	[66]
	Ultra-sounded pulp	1.33 ± 0.04	mg GAE/g f.w	[43]
	Waste (seeds, peel, and a minimal amount of pulp)	42.81 ± 2.23	mg GAE/100 g	[18]
	Fermented juice	1048.0 ± 8.0	mg GAE/L	[101]
	Freeze-dried peel	124.3 ± 87.3	mg GAE/g f.w	[21]
		1210.14 ± 225.4	mg GAE/100 g d.w	[64]
	Pasteurized pulp	53.9 ± 8.6	mg GAE/100 g f.w	[44]
	Hydromethanolic pulp extract	146.43	mg/100 g f.w	[102]
	Ethanolic seed extract	29.57	g GAE/100 g	[24]
	Apple nectar supplemented with freeze-dried pulp	0.554 ± 0.011	mg GAE/100 g f.w	[26]
	Frozen pulp	144.0 ± 0.06	mg GAE/100 g	[103]
	Dark chocolate with freeze-dried pulp	11.23 ± 1.10	mg GAE/g	[104]
	Pasteurized and combined with sucrose pulp	41.7 ± 2.5	mg GAE/100 g f.w	[45]
Flavonoids (total)	Pulp + peel	9.7	mg CE/g d.w	[12]
		1.25 ± 0.12	mg CE/g d.w	[14]
	Seeds	24.1	mg CE/g d.w	[12]
		43.73 ± 0.23	mg CE/g d.w	[14]
	Pulp	0.05 ± 0.0	mg CE/g	[97]
		2.55 ± 0.04	mg/100 g	[98]
	Ultra-sounded pulp	0.09 ± 0.0	mg CE/g f.w	[43]
	Waste (seeds, peel, and a minimal amount of pulp) aqueous extract	2.52 ± 0.25	mg Q/100 g	[18]
	Freeze-dried pulp	600.72 ± 22.25	mg CE/100 g d.w	[62]
	Apple nectar supplemented with freeze-dried pulp	1.537 ± 0.091	mg CE/100 g f.w	[26]
Carotenoids (total)	Freeze-dried pulp	44.29 ± 0.78	mg β-carotene eq/g d.w	[47]
		31.00 ± 0.22	µg β-carotene/100 g d.w	[62]
		806 ± 348	µg/100 g f.w	[21]
		62.85 ± 1.92	µg β-carotene/g	[96]
	Pulp + peel	0.9	mg/100 g f.w	[15]
	Freeze-dried peel	2484 ± 421	µg/100 g f.w	[21]
	Pulp	380.77 ± 7.13	µg β-carotene/100 g	[98]
Ascorbic acid	Freeze-dried pulp	92 ± 0.001	mg /100 g d.w	[47]
	Pulp	17.8 ± 0.3	µmol/g	[37]
		127.8	mg/100 g	[10]
		9.5 ± 0.8	mg/100 g f.w	[99]
		5.60 ± 0.01	mg/100 g	[98]
	Ultra-sounded pulp	13.67	mg/100 g	[43]
	Peel	184.8	mg/100 g	[10]
	Pulp + peel	8.3	mg/100 g f.w	[15]
	Pasteurized pulp	26.07 ± 0.1	mg/100 g f.w	[44]
	Hydromethanolic pulp extract	65.10	mg/100 g f.w	[102]
	Apple nectar supplemented with Freeze-dried pulp	24.95 ± 0.21	mg/100 mL	[26]
	Pasteurized and combined with sucrose pulp	32.3 ± 2.5	mg/100 g f.w	[45]
Phenolic compounds				
Caffeic acid	Freeze-dried pulp	66.49 ± 5.52	mg/100 g d.w	[47]
	Waste (seeds, peel, and a minimal amount of pulp) aqueous extract	0.46 ± 0.06	µg/g	[18]
	Hydromethanolic pulp extract	0.031 ± 0.02	mg/100 g f.w	[102]
Chlorogenic acid	Freeze-dried pulp	96.94 ± 1.83	mg/100 g d.w	[47]
	Hydromethanolic pulp extract	0.47 ± 0.09	mg/100 g f.w	[102]
Cinnamic acid	Freeze-dried pulp	126.58 ± 2.54	mg/100 g d.w	[47]
	Waste (seeds, peel, and a minimal amount of pulp) aqueous extract	44.61 ± 0.76	µg/g	[18]
	Hydromethanolic pulp extract	0.30 ± 0.08	mg/100 g f.w	[102]
Coumaric acid	Freeze-dried pulp	128.55 ± 3.48	mg/100 g d.w	[47]
	Waste (seeds, peel, and a minimal amount of pulp) aqueous extract	1.57 ± 0.15	µg/g	[18]
	Hydromethanolic pulp extract	0.02 ± 0.01	mg/100 g f.w	[102]
Gallic acid	Freeze-dried pulp	166.02 ± 3.39	mg/100 g d.w	[47]
	Waste (seeds, peel, and a minimal amount of pulp) aqueous extract	30.18 ± 5.47	µg/g	[18]
	Fermented juice	1048.0 ± 8.0	390 µM	[101]
	Hydromethanolic pulp extract	0.12 ± 0.02	mg/100 g f.w	[102]
Ferulic acid	Freeze-dried pulp	468.44 ± 10.10	mg/100 g d.w	[47]
	Waste (seeds, peel, and a minimal amount of pulp) aqueous extract	0.44 ± 0.02	µg/g	[18]
	Hydromethanolic pulp extract	0.96 ± 0.44	mg/100 g f.w	[102]
Quercetin	Freeze-dried pulp	699.75 ± 11.82	mg/100 g d.w	[47]
		5.16 ± 1.40	mg/100 g	[95]
		14.4 ± 0.2	mg/100 g d.w	[105]
	Waste (seeds, peel, and a minimal amount of pulp) aqueous extract	5.79 ± 0.17	µg/g	[18]
	Hydromethanolic pulp extract	0.67 ± 0.19	mg/100 g f.w	[102]
	Pulp	1.75 ± 0.03	mg/100 g f.w	[99]
Rutin	Freeze-dried pulp	413.33 ± 12.34	mg/100 g d.w	[47]
	Hydromethanolic pulp extract	0.40 ± 0.23	mg/100 g f.w	[102]
Eriodictyol	Freeze-dried pulp	575.15 ± 13.37	mg/100 g d.w	[47]
Myricetin	Freeze-dried pulp	17.0 ± 0.50	mg/100 g	[95]
Kaempherol	Freeze-dried pulp	3.70 ± 3.30	mg/100 g	[95]
		2.5 ± 0.1	mg/100 g d.w	[105]
	Pulp	0.30 ± 0.01	mg/100 g f.w	[99]
Vanillin	Waste (seeds, peel, and a minimal amount of pulp) aqueous extract	0.09 ± 0.0	µg/g	[18]
(-)-Epicatechin	Waste (seeds, peel, and a minimal amount of pulp) aqueous extract	18.05 ± 5.35	µg/g	[18]
	Dark chocolate with freeze-dried pulp	0.33 ± 0.04	mg/g	[104]
Ethyl gallate	Waste (seeds, peel, and a minimal amount of pulp) aqueous extract	0.37 ± 0.02	µg/g	[18]
Catechin	Hydromethanolic pulp extract	3.86 ± 1.83	mg/100 g f.w	[102]
	Dark chocolate with freeze-dried pulp	0.06 ± 0.01	mg/g	[104]
Carotenoids				
All-trans-β-carotene	Pulp + peel	2.10	µg/g	[85]
	Freeze-dried peel	143 ± 25	µg/g	[21]
	Freeze-dried pulp	44 ± 16	µg/g	[21]
All-trans-α-carotene	Pulp + peel	0.65	µg/g	[85]
	Freeze-dried peel	96 ± 20	µg/g	[21]
	Freeze-dried pulp	31 ± 14	µg/g	[21]
β-kryptoxanthin	Pulp + peel	2.44	µg/g	[85]
	Freeze-dried peel	153 ± 32	µg/g	[21]
	Freeze-dried pulp	92 ± 38	µg/g	[21]
9-cis-β-carotene	Pulp + peel	0.18	µg/g	[85]
13-cis-β-carotene	Pulp + peel	0.16	µg/g	[85]
15-cis-β-carotene	Pulp + peel	0.11	µg/g	[85]
Violaxanthin	Pulp + peel	1.04	µg/g	[85]
Lutein	Pulp + peel	1.60	µg/g	[85]
	Freeze-dried peel	756 ± 116	µg/g	[21]
	Freeze-dried pulp	154 ± 107	µg/g	[21]
Zeaxanthin	Pulp + peel	0.55	µg/g	[85]
Zeinoxanthin	Freeze-dried peel	187 ± 24	µg/g	[21]
	Freeze-dried pulp	54 ± 22	µg/g	[21]
Organic acids				
Malic acid	Pulp	244.5 ± 4.8	µmol/g	[37]
Succinic acid	Pulp	39.5 ± 3.2	µmol/g	[37]
Citric acid	Pulp	3.3 ± 0.1	µmol/g	[37]

d.w: dry weight; f.w: fresh weight; GAE: gallic acid equivalent; CE: catechin equivalent; TS: total solids; Q: quercetin.

### 3.3. Functional Properties of Arazá with Health Significance

Despite the wide variety of bioactive compounds that the fruit possesses, the reports associated with the biological activities of the fruit (and its parts) are pretty limited, with our study finding little information related to in vivo and in vitro assays of the different biological properties that it may possess, focusing specifically on antioxidant activity (which has been studied in most of the research reviewed for the present work) and others (in less depth and number of times), which are described next.

#### 3.3.1. Antioxidant Activity

Among the methodologies used for the determination of the antioxidant activity, the DPPH (2,2-diphenyl-1-picrylhydrazyl.), ABTS (2,2′-azino-bis(3-ethylbenzothiazoline-6-sulfonic acid)), ferric-reducing antioxidant power (FRAP), and oxygen radical absorbance capacity (ORAC) are the main ones reported for the fruit of Arazá (Table 2). The four methods involve the two mechanisms related to the neutralization of oxidant and radical species (electron and proton transfer). They are widely applied in research aimed at determining the antioxidant activity of foods. They are expressed as a function of different substances with known antioxidants (ascorbic acid, trolox, and butylated hydroxytoluene) [106].

It is considered that the antioxidant activity found in the different parts of the fruit is related to the content of the bioactive compounds (ascorbic acid, phenolic compounds, and carotenoids) present in them, such as in the case of freeze-dried Arazá pulp with two types of wall material (AAG and AMD), finding that the higher retention of phenolic compounds (phenolic acids and flavonoids) was obtained through the use of AMD, which was reflected in the antioxidant response measured through the DPPH, ABTS, and FRAP assays [47]. Consequently, the higher-than-expected bioavailability of the bioactive compound content after in vitro digestion assays is reflected in the antioxidant activity measured by both analytical methods. Here, the antioxidant activity of the seed and edible (pulp + peel) fractions decreased in the DPPH assay, from 8. 4 ± 0.2 to 0.3 ± 0.0 µmol TE/g and from 94.0 ± 6.3 to 16.8 ± 0.3 µmol TE/g for the edible and seed fraction, respectively.

In the same study, the antiradical capacity measured by the ABTS assay was significantly improved after the digestive process, finding values from 25.3 ± 1.6 to 70.4 ± 3.2 µmol TE/g in the edible fraction and 198 ± 10.8 to 216 ± 7.8 µmol TE/g in the seed. The same trend was observed in the antioxidant capacity measured in the ORAC assay, where the values of both fractions increased (values from 22.8 ± 1.9 to 72.9 ± 3.3 µmol TE/g in the edible fraction and 52.8 ± 3.8 to 92.0 ± 1.6 µmol TE/g in the seed). This behavior is attributed to the biochemical transformations the different substances undergo during the digestive processes, including releasing other compounds that can create a positive antioxidant environment that inactivates or decreases the incidence of pro-oxidant species [21]. 

The relationship between the observed antioxidant response and the content of specific metabolites is related and statistically significant, such as in the case of the content between the phenolic compounds and the antioxidant response observed by the ORAC test and the Trolox equivalent antioxidant capacity (TEAC), which have a significant correlation coefficient (r^2^ = 0.898 for both cases), which is also a product of the presence of other phytochemicals that may contribute to the oxidative potential of the fruit [97,100,105]. The same behavior was observed in the analysis of the edible and seed fractions of the fruit, where the high content of phenolic compounds in the seeds was reflected in the antioxidant capacity measured by the DPPH, ABTS, and ORAC assays, with these values being 23, 13, and 3 times higher than those of the edible fraction [14]. Similarly, in the study of the antioxidant activity of the pulp and peel of Arazá, it was observed that the antioxidant capacity measured through the ABTS, DPPH, and FRAP assays was correlated with the content of the metabolites present in each fraction, with the peel having the highest values in each of the assays. This is attributed to the content of carotenoids, (especially xanthophylls, *α*-carotene, and *β*-carotene), phenolic compounds, and vitamin C, which play a defensive role against environmental factors as well as fulfilling the biological demands of the plant [21].

In the same way that the state of ripening influences the content of the bioactive compounds, it also affects the antioxidant activity. In the study of the antioxidant capacity of the pulp and peel, a significant difference was observed for all the tests between the antioxidant capacity of the pulp and the peel, the latter being the one with the highest values between the two fractions [64]. In this same study, the mature state presented the most increased antioxidant activity according to the ABTS and DPPH assay for the epicarp. At the same time, in the mesocarp, it corresponded to the green state. Additionally, in the FRAP assay, the epicarp and mesocarp presented the highest values in the green state; these results were linearly correlated with the content of phenolic compounds in each of the maturity stages. For the epicarp, the r^2^ values for ABTS, DPPH, and FRAP were 0.946, 0.888, and 0.993, respectively, and for the mesocarp, 0.990, 0.985, and 0.998 for ABTS, DPPH, and FRAP, respectively [64]. Similarly, Galeano et al. [65] evaluated the ABTS and DPPH radical scavenging capacities expressed as the Trolox equivalent antioxidant capacity (TEAC) of Arazá pulp at different stages of ripening. The results indicated that for both assays, the advanced semi-ripe (¾) stage presented the highest TEAC for the DPPH radical (81.9 ± 3 µmol Trolox/100 g dry extract) and ABTS (241.8 ± 35 µmol Trolox/100 g dry extract) assays; in addition, it is observed that from the green ripening stage, there is an increase in the antioxidant activity until reaching the advanced semi-ripe (¾) stage, which is associated with the increased production of phenolic compounds, carotenoids, ascorbic acid, and other compounds, which are degraded after this stage due to the physiological processes of the plant such as biotic stress [107,108].

The antioxidant capacity of the Arazá by-products (seeds and peel) was also evaluated. In the corresponding study, the antioxidant capacity measured through the ORAC and FRAP assay was higher in the methanolic extracts (6791.07 ± 10.20 and 1380.74 ± 7.71 µmol TE/100 g, respectively) than in the aqueous extracts (3302.47 ± 3.11 and 798.92 ± 1.52 µmol TE/100 g, respectively) [18]. In contrast, the ABTS radical scavenging capacity was similar in both extracts (1553.39 ± 32.67 mg VCE/100 g for the aqueous extract and 1548.54 ± 42.3 mg VCE/100 g for the methanolic extract). In addition, the authors of the study correlate the content of the phenolic compounds with the antioxidant capacity measured between the different assays, finding correlation values for ABTS, FRAP, and ORAC of 0.971, 0.998, and 0.436 in the methanolic extract and in the aqueous extract of 0.916, 0.999, and 0.981, respectively [18]. 

The conditions used in the extraction process significantly influence the extraction performance, chemical composition, and stability. Due to this, optimizing the conditions is usually one of the main approaches in extracting bioactive compounds from plants [109]. In the evaluation of Arazá, seeds collected in the Colombian Andean region were extracted with different solvents [24]. It was observed that the DPPH radical uptake capacity was higher in the dichloromethane fraction (759.76 ± 17.1 mmol Trolox/100 g), followed by the residual hydroalcoholic fraction (710.73 ± 43.84 mmol Trolox/100 g) and the ethanol extract (759.76 ± 17.1 mmol Trolox/100 g), followed by the residual hydroalcoholic fraction (710.73 ± 43.84 mmol Trolox/100 g), and finally by that of the initial crude ethanolic extract (659.15 ± 19.07 mmol Trolox/100 g). In the same study, the ABTS radical scavenging capacity followed the same trend as in the previous assay, with the dichloromethane fraction being higher (5491.35 ± 86.91 mmol Trolox/100 g), followed by the hydroalcoholic residue (2275.40 ± 110.20 mmol Trolox/100 g), and finally the crude ethanolic extract (446.65 ± 2.82 mmol Trolox/100 g) [24]. The effect of the solvent used in the extraction process is also demonstrated in the study carried out by Borges et al. [15], where they evaluated the antioxidant capacity by the FRAP assay and the ABTS radical scavenging capacity of five different extracts with different approaches: methanolic extract (MeOH, focused on phenolic compounds), ethanolic extract (EtOH, focused on anthocyanins), acetonic extract (Acet, focused on carotenoids), aqueous extract (H_2_O, focused on ascorbic acid), and heptane extract (Hept, focused on tocopherols). The results indicated that the ethanolic extract (EtOH) presented the highest antioxidant capacity measured by the ORAC assay (54.3 µM TE/g f.w) and followed the order H_2_O (aqueous) > Acet (acetone) > Hept (heptane) > MeOH (methanolic). Regarding the ABTS radical scavenging capacity, the highest value also corresponded to the ethanolic assay (33.6 µM TE/g f.w) and followed the order Hept > MeOH > H_2_O > Acet > Hept > MeOH > H_2_O > Acet [15]. Although each extract had a special focus, the extracted compounds do not correspond exclusively to that substance, as they can be extracted using different solvents. Therefore, the antioxidant capacity observed measures all the extract’s components instead of the response of the isolated compounds. Hence, the different extracts represent the selectivity in which different bioactive compounds are extracted and stabilized according to the solvent’s type [110,111].

Due to the high perishability of the fruit after harvesting and to preserve its antioxidant activity, different transformations have been implemented, such as the application of heat treatments. In the study carried out by García-Reyes et al. [44], they evaluated the behavior of the antioxidant activity of Arazá puree (prepared from the pulp) when pasteurized at different temperatures (65, 73, and 80 °C). Despite an initial decrease in the antioxidant activity after heat treatment and two months of frozen storage, the antioxidant activity measured by the FRAP assay corresponded to 120, 123, and 106% of the initial antioxidant activity of the untreated puree at temperatures of 65, 73, and 80 °C, respectively. In the same study, when compared similarly, the ABTS radical scavenging capacity increased to 148, 170, and 155% of the antioxidant activity measured at day zero [44]. These changes are possibly attributed to the differential behavior of phenolic compounds during the pasteurization and frozen storage process, where they are degraded at different rates, may increase in concentration, or may be transformed to give rise to compounds with higher antioxidant activity. In another similar piece of research, Narváez-Cuenca et al. evaluated the effect of heat treatment and the addition of sucrose on preserving mashed Arazá pulp and on the antioxidant activity [45]. The results showed that heat treatment and sucrose addition control the degradation of vitamin C, phenolic compounds, and antioxidant activity (measured by ABTS radical assays, DPPH, and FRAP antioxidant capacity), increasing the chemical stability of these compounds in the matrix during frozen storage. Additionally, increases in the antioxidant activity were attributed to the production of non-enzymatic browning reactions due to the addition of sucrose and heating to low pH values [45].

Among other applications, the application of spray-drying on Arazá pulp led to the production of particles with a high content of phenolic compounds and antioxidant activity, with those produced in a 1:9 ratio (diluted pulp: maltodextrin) at 100 °C being the ones that presented the highest content of phenolic compounds (2934 ± 50 mg GAE/100 g d. w) and antioxidant activity measured by the ABTS radical scavenging capacity (247 ± 18 µmol Trolox/g d.w), DPPH (308 ± 7 g d.w/g DPPH), and FRAP assay (403 ± 6 µmol FeSO_4_/g d.w), as well as preserving these characteristics after the in vitro gastrointestinal digestion test and possessing desirable morphological characteristics and high thermal stability [66]. Similarly, other transformations and treatments have been applied to the fruit to preserve these characteristics, such as high-intensity ultrasound, in which the treatment with a higher intensity and duration increased the content of phenolic compounds and flavonoids by 13.53 and 33.33%, respectively; in addition, these changes are involved with the increase in the antioxidant activity measured through the DPPH and ABTS radical assays, where increases of 36.65 and 41% were observed about the control, respectively [43]. Modified atmospheres in packaging is another technique commonly used to preserve fruits and vegetables, guaranteeing a longer shelf life than untreated food. The research by Llerena et al. [62] investigated the effect of using a modified atmosphere on the physicochemical qualities of the Arazá. The results showed that the use of the modified atmosphere was able to preserve the antioxidant capacity (measured through the ABTS and DPPH radical capture assays), finding its maximum value on day four (574.49 and 1074.77 mg TE/100 g f.w for ABTS and DPPH, respectively).

On the other side, although the methods mentioned above are generally the most commonly used to express the antioxidant capacity, other methods also give insights into the antioxidant activity and the chemical composition of the substances responsible for this biological activity (such as a polarity and affinity to substrates). In the research carried out by Guimarães et al. [102], the antioxidant activity was evaluated in six different methods: the ABTS radical binding capacity, DPPH, β-carotene bleaching, thiobarbituric acid reactive substances (TBARs), reducing powder (ferricyanide), and phosmolybdenum complex, finding values of 5.84, 4.52, 2.94, 0.038, 8.09, and 7.81 mg butylated hydroxytoluene (BHT)/g f.w, respectively. 

Additionally, another method usually employed to evaluate the antioxidant capacity of foods is *β*-carotene bleaching, which uses an emulsified lipid substrate (linoleic acid); thus, this assay provides information on the ability of a compound or sample to protect biological molecules from oxidation, as would occur in the usual food matrix. The value reported for this method for Arazá pulp is 0.31 ± 0.01 µmol TE/g f.w. [99]. The importance of the variety of methods lies in the fact that antioxidants do not act independently, but through interactions with each other, and, given the complexity of food matrices, it is necessary to measure several methods as measuring them individually would result in a lower representation of the antioxidant behavior they may possess. Therefore, a greater number of assays will result in a better understanding of the nature of the sample being analyzed.

**Table 2:** Anti-radical and antioxidant capacity reported for Arazá.

Fruit Transformation	Scavenging Capacity				
	ABTS	DPPH	FRAP	ORAC	Reference
Freeze-dried pulp	n.r	n.r	1306.96 ± 6.22 μM Fe^2+^/g d.w	n.r	[47]
	n.r	IC50 = 0.69 ± 0.23 µg/mL	n.r	371.98 ± 11.50 µmol TE/100 g	[95]
	1.2 ± 0.3 µmol TE/g f.w	9.0 ± 8.6 µmol TE/g f.w	3.5 ± 0.9 µmol TE/g f.w	n.r	[21]
	n.r	46.2 µmol TE/g d.w	n.r	198.2 µmol TE/g d.w	[105]
	43.64 ± 2.14 µmol TE/g d.w	23.75 ± 1.89 µmol TE/g d.w	30.74 ± 3.79 µmol TE/g d.w 72.55 ± 6.31 µmol FeSO_4_/g d.w	n.r	[64]
	758.22 ± 5.01 µmol TE/g	392.10 ± 9.67 µmol TE/g	n.r	n.r	[96]
Pulp + peel	25.3 ± 1.6 TE/g d.w	8.4 ± 0.2 TE/g d.w	n.r	22.8 ± 0.2 µmol TE/g d.w	[12]
	12.46 ± 0.89 µmol TE/g d.w	43.15 ± 1.67 µmol TE/g d.w	n.r	29.42 ± 1.09 µmol TE/g d.w	[14]
	54.3 μM TE/g f.w	n.r	n.r	33.6 μM TE/g f.w	[15]
	40.9 μM TE/g	7.8 μM TE/g	n.r	n.r	[85]
Seeds	198.0 ± 10.8 TE/g d.w	94.0 ± 6.3 TE/g d.w	n.r	52.8 ± 6.3 µmol TE/g d.w	[12]
	n.r	n.r	78.8 ± 49.8 µmol TE/gTS	105.6 ± 12.3 µmol TE/gTS	[9]
	146.75 ± 2.44 µmol TE/g d.w	451.24 ± 2.87 µmol TE/g d.w	n.r	100.14 ± 2.55 µmol TE/g d.w	[14]
Pulp	5.5 ± 0.3 µmol TE/g	n.r	n.r	12.1 ± 0.4 µmol TE/g	[97]
	n.r	84.4 µmol TE/100 g d.w	n.r	97.7 µmol TE/100 g d.w	[10]
	n.r	n.r	165.4 ± 64.5 µmol TE/gTS	195.7 ± 4.2 µmol TE/gTS	[9]
	1209.72 ± 0.01 µmol TE/100 g	472.15 ± 0.40 µmol TE/100 g	1652.91 ± 0.72 µmol TE/100 g	n.r	[98]
	n.r	1.80 ± 0.05 µmol TE/g f.w	n.r	n.r	[99]
	2.11 ± 0.01 µmol TE/g	2.66 ± 0.06 µmol TE/g	8.64 ± 0.10 μmol Fe^2+^/g	n.r	[100]
	241.8 ± 35 µmol TE/g d.w	81.9 ± 3 µmol TE/g d.w	n.r	n.r	[65]
Peel	n.r	122.0 µmol TE/100 g d.w	n.r	130.5 µmol TE/100 g d.w	[10]
Spray-dried pulp	617 ± 15 µmol TE/g d.w	IC50 [54.59 mM] = 3.3 ± 0.1 mg d.w/mL 308 ± 7 g d.w/g DPPH	409 ± 8 µmol FeSO_4_/g d.w	n.r	[66]
Ultra-sounded pulp	5.38 ± 0.02 μM TE/g f.w	1.76 ± 0.02 μM TE/g f.w	n.r	3.22 ± 0.07 μM TE/g f.w	[43]
By-product (seeds, peel, and a minimal amount of pulp) aqueous extract	1553.39 ± 32.67 mg VCE/100 g	n.r	798.92 ± 1.52 µmol TE/100 g	3302.47 ± 3.11 µmol TE/100 g	[18]
Fermented juice	n.r	589.4 ± 5.8 μM TE	n.r	n.r	[101]
Freeze-dried peel	11.0 ± 5.3 µmol TE/g f.w	9.0 ± 8.6 µmol TE/g f.w	12.4 ± 7.7 µmol TE/g f.w	n.r	[21]
	171.88 ± 10.06 µmol TE/g d.w	125.35 ± 8.05 µmol TE/g d.w	64.49 ± 5.54 µmol TE/g d.w 170.21 ± 13.67 µmol FeSO_4_/g d.w	n.r	[64]
Pasteurized pulp	1.02 ± 0.15 mmol TE/100 g f.w	n.r	1.77 ± 0.15 mmol Fe^2+^/100 g f.w	n.r	[44]
	549.16 mg TE/100 g f.w	1075.67 mg TE/100 g f.w	n.r	n.r	[62]
Pulp pasteurized and combined with sucrose	8.4 ± 1.3 µmol TE/g f.w	3.9 ± 0.5 µmol TE/g f.w	9.7 ± 1.1 µmol TE/g f.w	n.r	[45]
Hydromethanolic pulp extract	4.52 mg BHTE/g f.w	5.84 mg BHTE/g f.w	8.09 mg BHTE/g f.w	n.r	[102]
Ethanolic seed extract	IC50 = 4.22 ± 0.02 mg/L 446.65 ± 2.82 mmol TE/100 g f.w	IC50 = 3.06 ± 0.09 mg/L 659.15 ± 19.07 mmol TE/100 g f.w	4.05 ± 0.36 µmol AAE/100 g f.w	274.22 ± 13.76 mmol TE/100 g f.w	[24]
Apple nectar supplemented with freeze-dried pulp	0.433 ± 0.013 µmol TE/g f.w	0.232 ± 0.006 µmol TE/g f.w	n.r	1.214 ± 0.087 µmol TE/g f.w	[26]
Frozen pulp	n.r	6.09 ± 0.29 µmol TE/100 g	n.r	n.r	[103]
Dark chocolate with freeze-dried pulp	n.r	IC50 = 5.19 ± 1.73 mg/mL	n.r	n.r	[112]

n.r: no reported; d.w: dry weight; f.w: fresh weight; TE: Trolox equivalent; TS: total solids; VCE: vitamin C equivalent; BHTE: butylated hydroxytoluene equivalent; AAE: ascorbic acid equivalent.

#### 3.3.2. Other Biological Properties 

As mentioned throughout the review, this fruit’s utilization and scientific research focused on its potential use are low, and this is reflected in the biological activities (leaving aside the antioxidant activity, which occupies, to a large extent, the most significant number of studies reviewed) that have been evaluated in the fruit. Therefore, this section synthesizes and describes the analyzed biological properties of Arazá.

Diabetes is a metabolic disorder of multiple origins characterized by hyperglycemia (elevated postprandial glucose levels in the bloodstream). It is considered one of the major non-communicable diseases (NCDs) and is of international importance as one of the leading causes of death worldwide [113]. The condition has been linked to unhealthy and highly sedentary lifestyles [114]. One of the current routes of action has focused on inhibiting the enzymes responsible for carbohydrate metabolism (*α*-amylase and *α*-glucosidase), which hydrolyze to small units of sugars (glucose, fructose, and galactose), which are subsequently absorbed in the intestine [115]. In the work carried out by Schmidt et al. [105], the inhibitory capacity of α-amylase and α-glucosidase of the pulp of Arazá was evaluated and the IC_50_ value for the inhibition of α-amylase was 3.3 mg d.w/mL and α-glucosidase was 2.4 mg d.w/mL. The same study showed that the content of phenolic compounds (especially flavonoids) was primarily related to the inhibitory capacity of both enzymes and that no relationship was observed between the antioxidant activity and inhibitory activity. In addition, it is essential to note that to establish the potential of a fruit as a therapeutic agent for the treatment of diabetes, it must have a moderate *α*-amylase inhibitory capacity and a high α-glucosidase inhibitory capacity, as shown by the Arazá fruit [100], highlighting the potential use of the fruit in future research related to glycemic control through diet, as this is the only report associated with this biological property and no in vivo trials have been carried out.

Inflammation, which is defined as an excess of inflammatory mediators including reactive oxygen species (ROS) and pro-inflammatory cytokines, has been treated using plant extracts in traditional medicine [116]. In the study carried out by Cintra-Soares et al. [63], where the anti-inflammatory capacity of the hydro-ethanolic extract of Arazá pulp was evaluated, a decrease in the activation of NF-KB cells in in vitro assays and in in vivo models (mice), where the flow of neutrophils was reduced by 42–55%, is similar in value to those observed in the positive control (dexamethasone) which is widely used in the control of chronic inflammatory diseases, thus highlighting the potential use of the fruit as it has health-promoting (anti-inflammatory) properties. In the same study, the scavenging capacity of reactive oxygen and nitrogen species (RNS) was evaluated for peroxide radicals (32.73 ± 1.50 µmol TE/g), superoxide anions (IC_50_ = 758.13 ± 18.30 µg/mL), nitric oxide (IC_50_ = 6.95 ± 0.81 µg/mL), and hypochlorous acid (IC_50_ = 14. 64 ± 3.07 µg/mL). These are species involved in oxidative stress, with their response being attributed to the presence of flavonoids, specifically quercetin, which is known for its potent antioxidant power and explains the marked ROS and RNS scavenging capacity [63].

The anti-proliferative activity of the ethanolic extract of the edible part of Arazá has also been studied. In the research carried out by Neri-Numa et al. [95], the effect on nine cell lines (U251, UACC-62, MCF-7, NCI-ADR/RES, 786-0, NCI-H460, PC-3, OVCAR-3, HT-29, and VERO) was studied, where the extract did not have a cytostatic effect on any of the cell lines evaluated. In addition, the same study evaluated the antigenotoxic and antimutagenic capacity of the extract, where a 300 mg extract/Kg concentration showed the most significant protective effect against damage to the genetic material. Similarly, another in vivo study was carried out on the consumption of Arazá pulp in rats, where the results indicated that no significant increases were observed in hematocrit, decreased levels of blood glucose, plasma cholesterol, glutamic pyruvic trans-aminase, creatinine, and urea levels [103]. 

Lastly, the anthelmintic activity of the Arazá seed extracts was evaluated, with the ethanolic crude extract being the one that presented the highest activity on gastrointestinal nematodes, with the lowest values of the inhibition of IC_50_ and a lethal concentration (LC_50_), unlike the dichloromethane fraction and the hydroalcoholic residue. This behavior is attributed to the extract’s constituents, with phenolic compounds of a tannic nature that, according to nature, have been shown to decrease the reproduction rate in parasitic species [24].

#### 3.3.3. Arazá Products and Further Processing

Due to their characteristic aroma, color, texture, and acidic taste, the products are especially suitable for food purposes, including wines [101], dehydrated pulps [116], micro-particles by spray-drying [66] and freeze-drying [46], supplemented nectars [26], jellies [21], sweets [11], non-fermented beverages [117], snacks [118], and chocolates [104]. Additionally, in the analysis of the database and experimental studies for the promotion of Latin America’s plant diversity for cosmetic use [9], Arazá had one of the highest values for peel protective activity; it has a high percentage of inhibition of enzymes such as tyrosinase as well as the reduction of fibroblast death in cells after being exposed to UVA radiation and induces an increase in the production of pro-collagen, thus bringing together a large number of desirable characteristics as an ingredient of plant origin with potential use in the cosmetics industry, with this being the first report for Arazá.

## 4. Conclusions and Perspectives 

Arazá (*Eugenia stipitata*) is a fruit with a high content of bioactive compounds (mainly ascorbic acid, phenolic compounds, and carotenoids) in its different fractions (pulp, seeds, and peel), as well as a varied composition of the same, with the diversity of the carotenoids and phenolic compounds it possesses being of particular importance, the same substances of which different health-promoting activities have been reported that can prevent or help to control non-communicable diseases. Antioxidant activity has been reported as one of the main biological properties present in the whole fruit, measured by different methods, and it is suggested that it may have an essential role in the control of oxidative stress, one of the leading causes of degenerative diseases and those associated with metabolic syndrome. However, it is agreed in the different statements of the reviewed studies that, unlike other fruits belonging to the Myrtaceae family, Arazá has been explored to a small extent, and its economic impact has not yet been adequately exploited. This is reflected in the number of research studies focused on its biological properties, where information is scarce and not very in-depth, in addition to the fact that in vitro and in vivo studies of other essential activities such as anti-obesity, anti-hypertensive, and others related to neurodegenerative diseases have not been evaluated, and remain unknown. 

Therefore, future studies related to Arazá must address approaches specialized in the potential of the fruit and its parts in treating and preventing NCDs. It is crucial to give special attention to the anti-diabetic, anti-hypertensive, anti-cancer, anti-inflammatory, and other possible effects related to the mechanisms of NCDs. Most of the studies also based their results on spectrophotometric assays and quantitative approaches based on standard substances. However, more than ever, it is necessary to use robust techniques such as LC-MS and GC-MS to identify specific compounds. Furthermore, new extraction methodologies must be used to avoid using conventional solvents, and more efficient methods are required to extend and enhance the composition and amount of compounds extracted.

Lastly, given the above, it is evident that research focused on this fruit is needed to expand and advance the number of applications it can have and identify other bioactive compounds present that can potentially be used in the treatment and prevention of NCDs, as well as in the food and pharmaceutical industries. In this way, the attractiveness of this fruit will increase, thereby contributing to its inclusion in the diet and the agronomical development of Amazonian fruits. 

## Data Availability

No new data were created or analyzed in this study. Data sharing is not applicable to this article.

## Supplementary Materials

The following supporting information can be downloaded at: https://www.mdpi.com/article/10.3390/foods13152310/s1, Figure S1. PRISMA 2020 flow diagram for new systematic reviews, which include searches of databases and registers only. The period covered goes from January 2000 to May 2023, where 315 were registered to end with 36 articles on the final review.
