# Supplementary material for: Arazá: Eugenia stipitata Mc Vaught as a Potential Functional Food
Source: Foods. 2024 Jul 23;13(15):2310. doi: 10.3390/foods13152310 (PMC11311875; doi:10.3390/foods13152310)
Supplement: Supplementary file 1 [file foods-13-02310-s001.zip › foods-3058642-supplementary.pdf]

Supplementary material

Figure S1. PRISMA 2020 flow diagram for new systematic reviews, which include searches of databases and registers only. The period covered goes from January 2000 to May 2023, where 315 were registered to end with 36 articles on the final review.

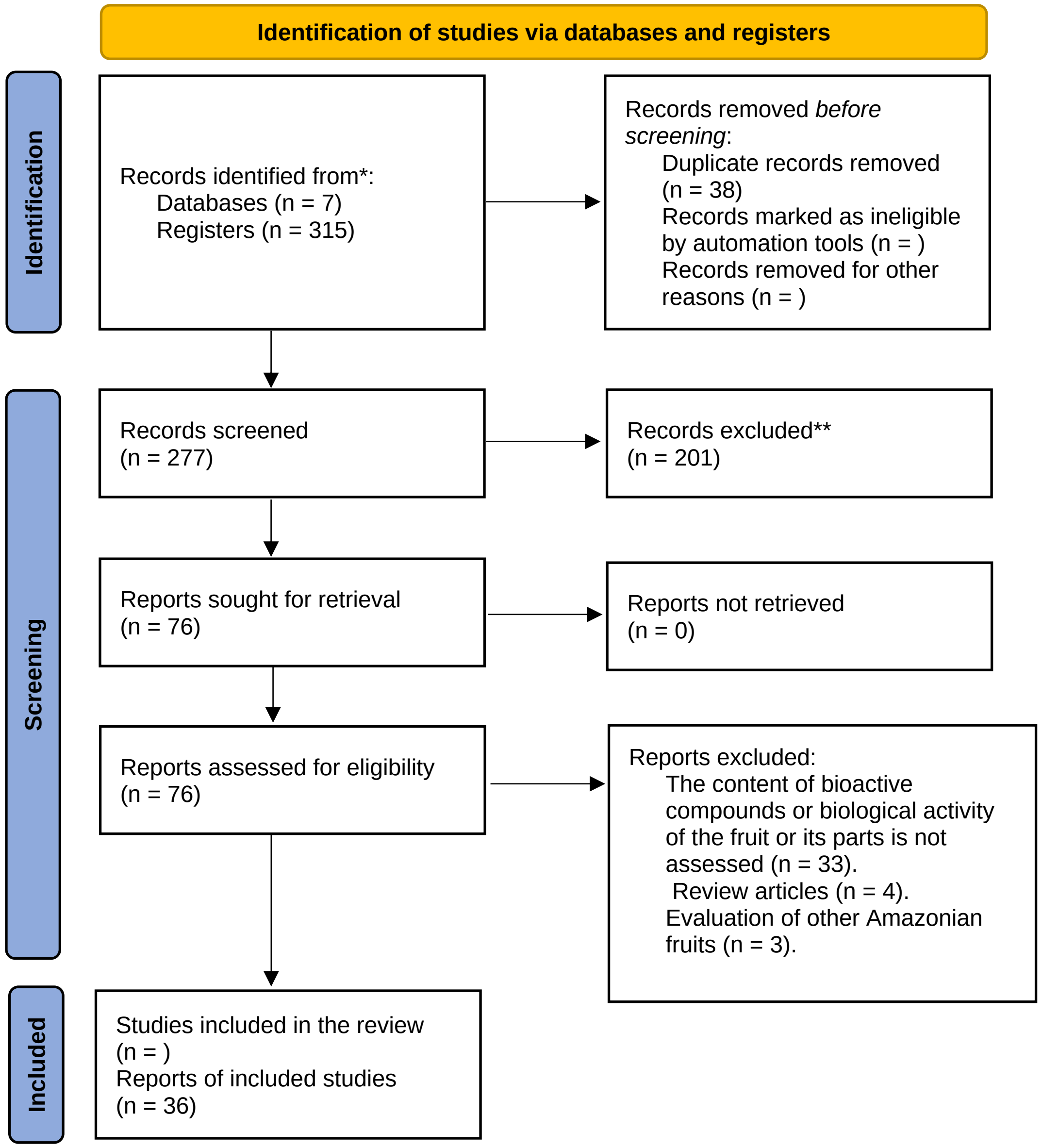

** No related to the evaluation of bioactive compounds, biological activity or extraction's performance. Other topics such as agronomy, soil chemistry, and plant growth.
